# Vitamin C Supplementation Slightly Improves Physical Activity Levels and Reduces Cold Incidence in Men with Marginal Vitamin C Status: A Randomized Controlled Trial

**DOI:** 10.3390/nu6072572

**Published:** 2014-07-09

**Authors:** Carol S. Johnston, Gillean M. Barkyoumb, Sara S. Schumacher

**Affiliations:** 1 School of Nutrition and Health Promotion, Arizona State University, 500 N. 3rd St., Phoenix, AZ 85004, USA; 2 Isagenix^®^ International LLC, 2225 S. Price Rd., Chandler, AZ 85286, USA; E-Mail: gillean.barkyoumb@isagenixcorp.com; 3 Cancer Treatment Centers of America^®^, 14200 W. Celebrate Life Way, Goodyear, AZ 85338, USA; E-Mail: sara.schumacher@ctca-hope.com

**Keywords:** vitamin C, physical activity, cold symptoms

## Abstract

The early indications of vitamin C deficiency are unremarkable (fatigue, malaise, depression) and may manifest as a reduced desire to be physically active; moreover, hypovitaminosis C may be associated with increased cold duration and severity. This study examined the impact of vitamin C on physical activity and respiratory tract infections during the peak of the cold season. Healthy non-smoking adult men (18–35 years; BMI < 34 kg/m^2^; plasma vitamin C < 45 µmol/L) received either 1000 mg of vitamin C daily (*n* = 15) or placebo (*n* = 13) in a randomized, double-blind, eight-week trial. All participants completed the Wisconsin Upper Respiratory Symptom Survey-21 daily and the Godin Leisure-Time Exercise Questionnaire weekly. In the final two weeks of the trial, the physical activity score rose modestly for the vitamin C group *vs.* placebo after adjusting for baseline values: +39.6% (95% CI [−4.5,83.7]; *p* = 0.10). The number of participants reporting cold episodes was 7 and 11 for the vitamin C and placebo groups respectively during the eight-week trial (RR = 0.55; 95% CI [0.33,0.94]; *p* = 0.04) and cold duration was reduced 59% in the vitamin C *versus* placebo groups (−3.2 days; 95% CI [−7.0,0.6]; *p* = 0.06). These data suggest measurable health advantages associated with vitamin C supplementation in a population with adequate-to-low vitamin C status.

## 1. Introduction

Vitamin C status of American adults is adequate with mean plasma concentrations ranging from 49 to 58 µmol/L for men and women, respectively [1,2]. However, 22% of U.S. adults are believed to have below adequate vitamin C status (plasma concentrations < 28 µmol/L), and about 6% of the adult population is classified as vitamin C deficient (<11 µmol/L) [2,3]. Non-supplementing men (20–49 years) are particularly at risk of poor vitamin C status [2]. While overt scurvy is reported occasionally in medical literature, poor vitamin C status is commonly undiagnosed since the early symptoms are nonspecific and unremarkable (e.g., fatigue, malaise, depression, and irritability) [4]. The fatigue and weakness of early scurvy have been attributed to defective carnitine production and the resultant reduction in fat oxidation [5,6,7]. Furthermore, vitamin C is a cosubstrate for dopamine-β-monooxygenase, which converts the neurotransmitter dopamine to norepinephrine [8]. The results of deficient neurotransmitter conversions may contribute to the depression and mood swings characteristic of early scurvy.

These feelings of fatigue and malaise may manifest as a reduced desire to be physically active. Early reports associated preclinical vitamin C deficiency with fatigue and aversion to exercise [9,10,11]. More recently, Pincemail *et al.* [12] evaluated nearly 900 healthy Belgian adults (40–60 years) and noted a 9% higher plasma vitamin C concentration in active individuals (those reporting physical activity two to three times a week) *versus* their inactive counterparts. However, there is little causal experimental evidence demonstrating an influence of vitamin C supplementation on daily physical activity.

Vitamin C may also reduce cold duration and severity [13]; however, this association continues to be highly debated. The reduction in cold duration has been linked to immune system enhancement by vitamin C [14]; whereas, the reduction in cold severity may be explained by the antihistamine property of vitamin C [15]. The 2013 Cochrane Review on vitamin C for the treatment of the common cold, encompassing twenty-nine trials with 11,306 research participants, did not observe a benefit of vitamin C supplementation on the incidence of colds in the general population, but a clear benefit of vitamin C supplementation for reducing the incidence of colds for individuals at times of extreme physical stress was noted (RR 0.48; 95% CI 0.35 to 0.64) [16].

The use of validated measures for assessing physical activity and respiratory tract symptoms is lacking in much of the published research on vitamin C supplementation and health outcomes. Furthermore, contradictions in the published research regarding benefits of vitamin C supplementation on common cold incidence and severity may be attributed in part to the vitamin C nutriture of the research participants at study entry [13]. The objective of this study was to determine the impact of vitamin C supplementation (1000 mg daily for eight weeks) on both physical activity levels and symptoms of upper respiratory tract infections in young men with marginal vitamin C status during the peak of the cold season (January to April). Validated measures, the Godin-Leisure Time Exercise Questionnaire and the Wisconsin Upper Respiratory Symptom Survey, were used to track change in physical activity and cold symptoms over the course of the trial.

## 2. Experimental Section

### 2.1. Participants

Healthy, nonsmoking men (*n* = 43; 18–35 years; BMI < 34 kg/m^2^) who did not engage in exercise training for competitive sports or use prescription medications were recruited from a large college campus in the Southwestern United States. Those regularly taking supplements or consuming foods fortified with over 60 mg vitamin C per serving were excluded. Qualifying volunteers provided written informed consent, and a postprandial venous blood sample (no food or drink with the exception of water for at least 5 h) was collected for vitamin C analysis. Individuals with plasma vitamin C concentrations less than 45 µmol/L were eligible to participate in the randomized study. These men (*n* = 30) were paired by age, BMI, and plasma vitamin C concentration and randomly assigned by a coin toss to the vitamin C group or the placebo group. The study was approved by the Institutional Review Board at Arizona State University and incorporates theses research conducted by Masters Students at Arizona State University [17,18]. This trial was not registered at an International Committee of Medical Journal Editors registry.

### 2.2. Study Design

This randomized, double-blind, placebo-controlled study followed a parallel arm design and lasted eight weeks. Participants were instructed to restrict all juice or fruit-flavored drinks during the study period, and to ingest two capsules daily in a divided dose (morning and evening). Vitamin C capsules (500 mg vitamin C per capsule, Twinlab C-500 CAPS, American Fork, UT, USA) were identical in appearance to the placebo capsules that contained white flour. All capsules were packaged in re-sealable storage bags. The randomization was conducted by the lead investigator who did not have contact with participants or conduct data entry or blood analyses. Participants were provided a booklet at the start of the study that contained the Wisconsin Upper Respiratory Symptom Survey-21, the Godin Leisure-Time Exercise Questionnaire, and a short food frequency measure. Participants completed the symptom survey daily, and the exercise and food frequency measures were completed weekly. The booklets contained materials to cover a 4-week time frame. At study week 4, participants received a second booklet to complete during study weeks 5–8. Diet quality was assessed at baseline and week 8 using the Rapid Eating and Activity Assessment for Patients tool. Fasting venous blood samples were collected at study weeks 4 and 8 for vitamin C and histamine analyses. Body and fat mass were measured using a calibrated bioelectric impedance scale (TBF-300A Body Composition Analyzer, Tanita Corporation of America, Inc., Arlington Heights, IL, USA), and height was measured using a mechanical stadiometer. The study was conducted January through April 2011, and data were unblinded once blood analyses and data entry were complete.

### 2.3. Survey Measures

The Wisconsin Upper Respiratory Symptom Survey-21 is a validated measure to assess the symptoms and impact of acute upper respiratory tract infections (presumed to be the common cold) [19]. Cold symptom severity (10 questions) and the impact of cold symptoms on daily living (9 questions) were assessed daily on a 7-point scale from “very mildly” to “severely”. Question scores were summed for each category to calculate the total symptom severity score and the total “impact of cold on daily living” score. For this research, a cold episode was defined as a daily score of 5 or greater for the symptom severity category, indicating either (1) the presence of five different “very mild” cold symptoms; (2) the presence of several different cold symptoms, some mild and some more moderate in severity; or (3) the presence of a single cold symptom at least moderate in severity. The duration of a cold was based on a daily score of 5 or greater on consecutive days. When two episodes of sickness were separated by one to three days when reported scores were <5, the episode was considered to be a single cold. Since some participants reported cold episodes on the first few days of the study, only cold episodes initiating after the 5th day of the study were counted. Cold duration (days), cold severity score (daily average), and the “impact of cold on daily living” score (daily average) were calculated for the reported cold episodes.

Leisure-time physical activity levels were assessed using the Godin-Leisure Time Exercise Questionnaire that was designed to estimate the energy cost of activities, or metabolic equivalents (METS) [20]. This physical activity questionnaire is considered highly reliable and has been validated with objective measures of physical activity such as accelerometers and heart rate monitors [20,21,22]. The times per week that mild, moderate, or strenuous exercise were engaged were multiplied by 3, 5, and 9 METS respectively and summed to estimate total METS (kcal·kg^−1^·week^−1^). For reporting purposes, scores for METS were averaged every two weeks during the trial. 

The Rapid Eating and Activity Assessment for Patients (REAPS) tool was designed to rapidly assess diets according to the U.S. Dietary Guidelines [23]. The REAPS demonstrated strong agreement with the Block Semi Quantitative Food Frequency Questionnaire for fruits, vegetables, milk, and fat consumption [24]. The food frequency measure was composed of 10 fruit, vegetable, and fortified food categories that represent the common sources of dietary vitamin C. Portion size and weekly serving frequency were recorded and used to estimate daily dietary vitamin C intake.

### 2.4. Biosample Analyses

A plasma aliquot was immediately mixed with equal volumes of ice-cold 10% trichloroacetic acid, centrifuged at 3500× *g* for 20 min at 0 °C, and the supernatant stored at −80 °C until analysis for vitamin C using the 2,4-dinitrophenylhydrazine colorimetric method [25] with inter-analyses coefficients of variation averaging <2.8%. This analysis highly correlates with HPLC methodology when fasting blood samples are used [26]. For histamine analysis, plasma was treated with an acylation reagent, and the acylated histamine was quantified using a commercial enzyme immunoassay kit (ALPCO Immunoassays, Salem, NH, USA).

### 2.5. Statistical Analysis

Values are expressed as the mean ± SD, and all data analyses were conducted using PASW Statistics 19.0 (Predictive Analytics SoftWare Statistics package, IBM, 2009). Based on pilot data, a sample of 15 participants per group provided 75% power to detect an 11 µmol/L difference in plasma vitamin C. Differences between means were examined using the nonparametric Mann-Whitney *U* test. Average change in outcome measures, 95% CI, and partial eta squared effect size (η2partial) were calculated using Univariate Analyses. Effect size describes the magnitude of the difference between variables and are interpreted as small (<0.02), medium (0.13) and large (>0.26). Pearson correlation was used to examine relationships between variables, and the rate ratio (RR) is reported for cold outcomes in the vitamin C supplemented group in comparison to the control group. For all analyses, significance was set at *p* ≤ 0.05.

## 3. Results

Participants were apparently healthy men. Plasma vitamin C concentrations were below adequate (<28 µmol/L) for 14 of 30 participants, and two participants were vitamin C deficient (plasma vitamin C < 11 µmol/L). Participants were paired by age, BMI and plasma vitamin C concentration and randomly assigned to the vitamin C (*n* = 15) or placebo (*n* = 15) groups. One participant randomized to the placebo group was non-compliant with the capsule protocol and removed from the trial after several weeks. An additional participant (also from the placebo group) reported a severe cold that lasted for the first 24 days of the trial, and his data were not entered into the analyses leaving 15 participants in the vitamin C group and 13 participants in the placebo group. Participants did not report any side effects with capsule ingestion. Baseline characteristics did not vary between groups for those participants who completed the trial with the exception of physical activity level (Table 1). At baseline vitamin C concentrations were inversely correlated with body weight (*r* = −0.597; *p* = 0.002), BMI (*r* = −0.523; *p* = 0.004), and body fat percentage (*r* = −0.623; *p* < 0.001). Aside from correlations between the adiposity measures, no other significant correlations were noted among baseline characteristics.

**Table 1:** Baseline characteristics by group *.

Characteristic	Vitamin C (*n* = 15)	Placebo (*n* = 13)
Age (year)	23.0 ± 3.1	23.2 ± 4.3
Weight (kg)	83.0 ± 9.1	81.6 ± 10.3
BMI (kg/m^2^)	24.5 ± 3.9	26.0 ± 3.9
Body fat (%)	17.5 ± 6.4	20.7 ± 6.5
Dietary vitamin C (mg/day)	93 ± 53	104 ± 45
Diet quality score	31.1 ± 3.1	31.2 ± 3.2
METS (kcal·kg^−1^·week^−^^1^)	57 ± 24	38 ± 22
Plasma vitamin C (µmol/L)	30.2 ± 8.5	29.1 ± 9.3

* Values are mean ± SD. There were no significant differences between groups with the exception of METS (*p* = 0.03, Mann-Whitney *U* test).

Fasting plasma vitamin C concentrations were raised significantly for the vitamin C group *versus* the placebo group at week 4 (41.3 ± 10.9 *versus* 30.8 ± 11.4 µmol/L; *p* = 0.02) and at week 8 (42.1 ± 10.4 *versus* 33.9 ± 10.9 µmol/L; *p* = 0.05). Diet quality did not change significantly between groups at week 8 (REAPS scores, 32.0 ± 3.4 and 30.5 ± 4.7 for the vitamin C and placebo groups respectively); furthermore, dietary vitamin C did not differ significantly between groups during the trial (average weekly intake, 93 ± 36 and 103 ± 53 mg/day for the vitamin C and placebo groups respectively).

Physical activity increased slightly in the vitamin C group compared to the placebo group in the first four weeks of the trial (+6.2% to +7.5%; *p* ≥ 0.7); however, at weeks 5–6 and weeks 7–8, physical activity increase more markedly in the vitamin C *versus* placebo groups (+21.2%, 95% CI [−23.7, 66.1], *p* = 0.3 and +39.6%, 95% CI [−4.5, 83.7], *p* = 0.10, respectively) (Table 2).

**Table 2:** The energy cost of activities, or metabolic equivalents (METS; kcal·kg^−1^·week^−1^) in participants ingesting vitamin C (1000 mg) or placebo daily for eight weeks. The data are averaged at two-week intervals and presented as the mean value; the percent increase from baseline with 95% CI; and the treatment effect *.

METS	Vitamin C (*n* = 15)	Placebo (*n* = 13)	Treatment effect	*p* [η2]
Week 1–2				
Mean ± SD	68 ± 32	44 ± 25		
Average increase	28.8%	21.3%	+7.5%	0.7
[95% CI]	[−3.7, 61.3]	[−13.6, 56.2]	[−40.1, 55.2]	[0.00]
Week 3–4				
Mean ± SD	66 ± 27	42 ± 25		
Average increase	21.0%	14.8%	+6.2%	0.8
[95% CI]	[−3.9, 45.8]	[−11.9, 41.5]	[−30.3, 42.6]	[0.01]
Week 5–6				
Mean ± SD	84 ± 41	46 ± 25		
Average increase	51.4%	30.1%	+21.2%	0.3
[95% CI]	[20.8, 82.0]	[−2.7, 63.0]	[−23.7, 66.1]	[0.04]
Week 7–8				
Mean ± SD	86 ± 39	43 ± 20		
Average increase	60.2%	20.5%	+39.6%	0.10
[95% CI]	[30.1, 90.2]	[−11.7, 52.8]	[−4.5, 83.7]	[0.12]

* *p* for Univariate analysis; η2 = partial eta squared effect size.

The number of participants reporting cold episodes was 7 and 11 for the vitamin C supplemented *versus* placebo groups respectively during the eight-week trial (RR = 0.55, 95% CI [0.33, 0.94], *p* = 0.04) (Table 3). The total number of reported colds was 12 and 17 for the vitamin C *versus* placebo groups (RR = 0.66, 95% CI [0.28, 1.55], *p* = 0.11). Cold duration was reduced 59% in the vitamin C *versus* placebo groups (−3.2 days, 95% CI [−7.0, 0.6]; *p* = 0.06). Cold severity scores and “impact of cold on daily living” scores did not differ between groups (Table 2). The overall mean symptom severity score of participants was not related to the average change in weekly METS during the 8-week trial.

**Table 3:** Cold outcomes in participants ingesting 1000 mg vitamin C *versus* placebo daily for eight weeks *.

	*n*	Participants with colds	Total number of colds	Cold duration, days (mean ± SD)	Cold severity score (mean ± SD)	Impact of cold on daily living score (mean ± SD)
Vitamin C	15	7	12	2.2 ± 1.4	10.7 ± 6.0	2.6 ± 1.6
Placebo	13	11	17	5.4 ± 4.5	18.6 ± 17.5	2.4 ± 2.1
RR or change 95% CI		0.55 ^a^ [0.33, 0.94]	0.66 ^a^ [0.28, 1.55]	−3.2 ^b^ [−7.0, 0.6]	−7.9 ^b^ [−22.5, 6.7]	0.2 ^b^ [−1.8, 2.2]
Effect size ^c^				0.170	0.075	0.003
*p* value ^d^		0.04	0.11	0.06	0.54	0.72

* Severity score: daily average total symptom score (ten items scored individually from 1 “very mild” to 7 “severe”: runny nose, plugged nose, sneezing, sore throat, scratchy throat, cough, hoarseness, head congestion chest congestion, feeling tired) for cold episodes. Impact of cold on daily living score: daily average total score (nine items scored individually from 1 “very mildly” to 7 “severely”: think clearly, sleep well, breathe easily, walk/climb stairs/exercise, accomplish daily activities, work outside the home, work inside the home, interact with others, live your personal life) for cold episodes. ^a^ Rate ratio; ^b^ treatment effect: average change; ^c^ partial eta squared; ^d^ Mann-Whitney *U* test or Chi Square test for “Participants with colds”.

Histamine concentrations did not correlate significantly with plasma vitamin C concentrations at week 8 of the study (*r* = −0.246, *p* = 0.207), and histamine concentrations were not impacted by eight weeks of vitamin C supplementation (0.667 ± 0.628 and 0.356 ± 0.2427 ng/mL at baseline *versus* 0.639 ± 0.277 and 0.431 ± 0.298 ng/mL at week 8 for the vitamin C and placebo groups respectively; *p* = 0.8 for eight-week change by group). However, histamine concentrations at week 8 were significantly correlated to the average daily cold severity score (*r* = 0.653, *p* = 0.003). Vitamin C concentrations at week 4 and at week 8 were also correlated to the average daily cold severity score (*r* = −0.711, *p* = 0.001 and *r* = −0.618, *p* = 0.006, respectively).

## 4. Discussion

These data demonstrate a measureable benefit of vitamin C supplementation for reducing cold episodes in young men with low to average vitamin C status. In addition, the data suggest a modest benefit of vitamin C supplementation for enhancing weekly activity levels in young men. Several large, cross-sectional investigations support a link between vitamin C status and physical activity and corroborate early reports that associated preclinical vitamin C deficiency with fatigue and aversion to exercise [9,10,11,27]. However, there is little causal experimental evidence demonstrating the influence of vitamin C supplementation on daily physical activity. Placebo-controlled intervention studies have documented increased work efficiency during exercise [7] and reduced perception of effort during exercise [28] in vitamin C supplementing untrained adults—characteristics that could possibly inspire physical activity. The role of vitamin C in promoting physical activity may relate to its antioxidant properties since oxidative stress is related to fatigue [29,30]. Vitamin C also possesses neuroprotective properties and influences the brain’s oxidative fuel supply [31], processes that may influence a sense of wellbeing.

The marked reduction in cold incidence and cold duration in young men ingesting vitamin C supplements adds to a large, and controversial, literature on vitamin C and prevention of the common cold. In 1975 Chalmers reviewed eight trials and concluded that there was no benefit of vitamin C supplementation for reducing the incidence or severity of colds [32]; however, critics reanalyzed these data and concluded that, although the incidence of colds was not impacted, vitamin C supplementation did significantly reduce the duration of colds by 21% [33]. Recent systematic reviews on vitamin C and the common cold agree that although the incidence of infections does not appear to be impacted by vitamin C, vitamin C supplementation is associated with reduced severity and duration of colds [34,35]. The most recent Cochrane review on vitamin C and colds examined 29 placebo-controlled trials that supplemented at least 200 mg vitamin C daily and concluded that vitamin C did not reduce the incidence of colds in the general population; however, in stressed populations, such as marathon runners and soldiers, cold incidence was reduced 52% [16]. Furthermore, the review showed that the duration of colds was shortened significantly, 8% and 14% for adults and children respectively. Based on the evidence to date, it may be that only populations with low vitamin C status, or those experiencing extreme physical exertion or cold stress, may experience anti-cold benefits of supplemental vitamin C.

A specific anti-cold mechanism of vitamin C has not been elucidated but may relate to immune-enhancement orchestrated by vitamin C such as improved natural killer cell activities [36] or improved lymphocyte proliferation or chemotaxis [37,38]. Although cold duration was impacted by vitamin C supplementation in the present trial, vitamin C did not reduce cold symptom severity as has been reported in the systematic reviews. Both the antioxidant and antihistamine properties of vitamin C have been implicated in cold symptom relief since oxidative stress and histamine contribute to cold severity [39,40]. Interestingly, histamine and vitamin C concentrations were correlated with cold symptom severity during the eight-week trial; however, vitamin C supplementation did not alter circulating histamine levels in this sample.

This study is limited by the small sample size. Although participants were screened for purposeful exercise and participation in competitive sports, routine physical activity levels did differ significantly between groups at baseline limiting the interpretation of the physical activity outcome data. A common cold episode was arbitrarily defined in this report, and these data may not be easily compared between reports. By design, this study was conducted in winter months and included only men with low-to-adequate vitamin C status; hence, generalizability of these results can only be extended to similar populations and season.

## 5. Conclusions

These data suggest that measurable health advantages are associated with vitamin C supplementation in men with adequate-to-low vitamin C status. This simple dietary strategy to promote physical activity and physical health merits further research and the consideration of health practitioners.

## References

[1] Pfeiffer C.M., Sternberg M.R., Schleicher R.L., Rybak M.E. (2013). Dietary supplement use and smoking are important correlates of biomarkers of water-soluble vitamin status after adjusting for sociodemographic and lifestyle variables in a representative sample of U.S. adults. J. Nutr..

[2] Schleicher R.L., Carroll M.D., Ford E.S., Lacher D.A. (2009). Serum vitamin C and the prevalence of vitamin C deficiency in the United States: 2003–2004 National Health and Nutrition Examination Survey (NHANES). Am. J. Clin. Nutr..

[3] Pfeiffer C.M., Sternberg M.R., Schleicher R.L., Haynes B.M., Rybak M.E., Pirkle J.L. (2013). The CDC’s Second National Report on Biochemical Indicators of Diet and Nutrition in the U.S. Population is a valuable tool for researchers and policy makers. J. Nutr..

[4] Ben-Zvi G.T., Tidman M.J. (2012). Be vigilant for scurvy in high-risk groups. Practitioner.

[5] Hughes R.E., Hurley R.J., Jones E. (1980). Dietary ascorbic acid and muscle carnitine (beta-OH-gamma-(trimethylamino) butyric acid) in guinea-pigs. Br. J. Nutr..

[6] Ha T.Y., Otsuka M., Arakawa N. (1994). Ascorbate indirectly stimulates fatty acid utilization in primary cultured guinea pig hepatocytes by enhancing carnitine synthesis. J. Nutr..

[7] Johnston C.S., Corte C., Swan P.D. (2006). Marginal vitamin C status is associated with reduced fat oxidation during submaximal exercise in young adults. Nutr. Metab. (Lond.).

[8] Wimalasena K., Wimalasena D.S. (1995). The reduction of membrane-bound dopamine beta-monooxygenase in resealed chromaffin granule ghosts. Is intragranular ascorbic acid a mediator for extragranular reducing equivalents?. J. Biol. Chem..

[9] Crandon J.H., Lund C.G., Dill D.B. (1940). Experimental human scurvy. N. Engl. J. Med..

[10] Hodges R.E., Baker E.M., Hood J., Sauberlich H.E., March S.C. (1969). Experimental scurvy in man. Am. J. Clin. Nutr..

[11] Cheraskin E., Ringsdorf W.M., Medford F.H. (1976). Daily vitamin C consumption and fatigability. J. Am. Geriatr. Soc..

[12] Pincemail J., Vanbelle S., Degrune F., Cheramy-Bien J.P., Charlier C., Chapelle J.P., Giet D., Collette G., Albert A., Defraigne J.O. (2011). Lifestyle behaviours and plasma vitamin C and β-carotene levels from the ELAN population (Liège, Belgium). J. Nutr. Metab..

[13] Hemilä H. (1997). Vitamin C intake and susceptibility to the common cold. Br. J. Nutr..

[14] Wintergerst E.S., Maggini S., Hornig D.H. (2006). Immune-enhancing role of vitamin C and zinc and effect on clinical conditions. Ann. Nutr. Metab..

[15] Uchida K., Mitsui M., Kawakishi S. (1989). Monooxygenation of *N*-acetylhistamine mediated by l-ascorbate. Biochim. Biophys. Acta.

[16] Hemilä H., Chalker E. (2013). Vitamin C for preventing and treating the common cold. Cochrane Database Syst. Rev..

[17] Schumacher S. Plasma Vitamin C Supplementation and Physical Activity in Young Men. Master’s thesis.

[18] Osterday G. Vitamin C and Treating the Common Cold. Master’s thesis.

[19] Barrett B., Brown R.L., Mundt M.P., Thomas G.R., Barlow S.K., Highstrom A.D., Bahrainian M. (2009). Validation of a short form Wisconsin Upper Respiratory Symptom Survey (WURSS-21). Health Qual. Life Outcomes.

[20] Godin G., Shephard R.J. (1985). A simple method to assess exercise behavior in the community. Can. J. Appl. Sport Sci..

[21] Sallis J.F., Buono M.J., Roby J.J., Micale F.G., Nelson J.A. (1993). Seven-day recall and other physical activity self-reports in children and adolescents. Med. Sci. Sports Exerc..

[22] Miller D.J., Freedson P.S., Kline G.M. (1994). Comparison of activity levels using the Caltrac accelerometer and five questionnaires. Med. Sci. Sports Exerc..

[23] Gans K.M., Ross E., Barner C.W., Wylie-Rosett J., McMurray J., Eaton C. (2003). REAP and WAVE: New tools to rapidly assess/discuss nutrition with patients. J. Nutr..

[24] Gans K.M., Risica P.M., Wylie-Rosett J., Ross E.M., Strolla L.O., McMurray J., Eaton C.B. (2006). Development and evaluation of the nutrition component of the Rapid Eating and Activity Assessment for Patients (REAP): A new tool for primary care providers. J. Nutr. Educ. Behav..

[25] Omaye S.T., Turnbull J.D., Sauberlich H.E. (1979). Selected methods for the determination of ascorbic acid in animal cells, tissues, and fluids. Methods Enzymol..

[26] Sauberlich H.E., Kretsch M.J., Taylor P.C., Johnson H.L., Skala J.H. (1989). Ascorbic acid and erythorbic acid metabolism in nonpregnant women. Am. J. Clin. Nutr..

[27] Ottevaere C., Huybrechts I., Béghin L., Cuenca-Garcia M., de Bourdeaudhuij I., Gottrand F., Hagströmer M., Kafatos A., Le Donne C., Moreno L.A. (2011). Relationship between self-reported dietary intake and physical activity levels among adolescents: The HELENA study. Int. J. Behav. Nutr. Phys. Act..

[28] Huck C.J., Johnston C.S., Beezhold B.L., Swan P.D. (2013). Vitamin C status and perception of effort during exercise in obese adults adhering to a calorie-reduced diet. Nutrition.

[29] Norheim K.B., Jonsson G., Omdal R. (2011). Biological mechanisms of chronic fatigue. Rheumatology (Oxf.).

[30] Van Zuiden M., Kavelaars A., Amarouchi K., Maas M., Vermetten E., Geuze E., Heijnen C.J. (2012). IL-1β reactivity and the development of severe fatigue after military deployment: A longitudinal study. J. Neuroinflammation.

[31] Castro M.A., Beltrán F.A., Brauchi S., Concha I.I. (2009). A metabolic switch in brain: Glucose and lactate metabolism modulation by ascorbic acid. J. Neurochem..

[32] Chalmers T.C. (1975). Effects of ascorbic acid on the common cold. An evaluation of the evidence. Am. J. Med..

[33] Hemilä H., Herman Z.S. (1995). Vitamin C and the common cold: A retrospective analysis of Chalmers’ review. J. Am. Coll. Nutr..

[34] Nahas R., Balla A. (2011). Complementary and alternative medicine for prevention and treatment of the common cold. Can. Fam. Physician.

[35] Heimer K.A., Hart A.M., Martin L.G., Rubio-Wallace S. (2009). Examining the evidence for the use of vitamin C in the prophylaxis and treatment of the common cold. J. Am. Acad. Nurse Pract..

[36] Heuser G., Vojdani A. (1997). Enhancement of natural killer cell activity and T and B cell function by buffered vitamin C in patients exposed to toxic chemicals: The role of protein kinase-C. Immunopharmacol. Immunotoxicol..

[37] Boxer L.A., Vanderbilt B., Bonsib S., Jersild R., Yang H.H., Baehner R.L. (1979). Enhancement of chemotactic response and microtubule assembly in human leukocytes by ascorbic acid. J. Cell Physiol..

[38] Anderson R., Oosthuizen R., Maritz R., Theron A., van Rensburg A.J. (1980). The effects of increasing weekly doses of ascorbate on certain cellular and humoral immune functions in normal volunteers. Am. J. Clin. Nutr..

[39] Garofalo R.P., Kolli D, Casola A. (2013). Respiratory syncytial virus infection: Mechanisms of redox control and novel therapeutic opportunities. Antioxid. Redox Signal..

[40] Johnston C.S. (1996). The antihistamine action of ascorbic acid. Subcell Biochem..

